# Anesthetic Management of a Pregnant Patient With Ehlers-Danlos Syndrome Undergoing Elective Cesarean Delivery: A Case Report

**DOI:** 10.7759/cureus.89400

**Published:** 2025-08-05

**Authors:** Yahya M Aljuba, Daniel Shatalin, Alexander Ronenson, Aharon Grenader, Alexander Ioscovich

**Affiliations:** 1 Department of Anesthesiology, Perioperative Medicine and Pain Treatment, Shaare Zedek Medical Center (Affiliated with the Hebrew University of Jerusalem), Jerusalem, ISR; 2 Department of Anaesthesiology, Hebrew University of Jerusalem, Faculty of Medicine, Jerusalem, ISR

**Keywords:** cesarean section (cs), ehlers-danlos syndrome, multidisciplinary approach, neuraxial anesthesia, obstetric anesthesia

## Abstract

Pregnancy in women with Ehlers-Danlos syndrome (EDS) carries elevated risks, including prematurity, hemorrhage, and maternal morbidity, posing significant anesthetic challenges. We present the case of a 36-year-old woman with classical EDS (cEDS) and multiple comorbidities, including postural orthostatic tachycardia syndrome, bronchial asthma, congenital adrenal hypoplasia, and chronic venous thrombosis, who underwent an elective cesarean section. A multidisciplinary team developed a comprehensive perioperative plan featuring ultrasound-guided spinal anesthesia, extended post-anesthesia observation, and coordinated recommendations from cardiology, hematology, endocrinology, pulmonology, and other specialties. This report emphasizes the complexity of anesthetic care in EDS pregnancies and underscores the need to tailor management to the specific EDS subtype and individual patient profile.

## Introduction

Ehlers-Danlos syndromes (EDS) represent a heterogeneous group of heritable connective tissue disorders characterized by varying degrees of joint hypermobility, skin hyperextensibility, tissue fragility [1], and widespread manifestations in the skin, ligaments and joints, blood vessels, and internal organs [1,2]. Pregnancies in women with EDS are associated with cervical incompetence, preterm labor, antepartum hemorrhage, placenta previa, and maternal death [2]. Pregnant women with EDS are more likely to require cesarean delivery. In addition, these patients often experience prolonged postpartum hospitalization [3]. These patients present unique anesthetic challenges due to the variability in tissue fragility, hypermobility, and potential cardiovascular involvement [4,5]. Considering the multisystemic eﬀects of EDS on the body, it is not surprising that the condition complicates pregnancy and delivery. Tailoring anesthetic management to the individual patient's clinical presentation and comorbidities is critical to ensuring safe outcomes. This article presents a detailed case-based approach to perioperative care in a pregnant patient with EDS.

## Case presentation

A 36-year-old pregnant woman (G3P2A0), classified as ASA III with a BMI of 31.2 kg/m², presented at 36+6 weeks’ gestation for an elective cesarean delivery (CD) with bilateral salpingo-oophorectomy. She was diagnosed with classical Ehlers-Danlos Syndrome (cEDS) at age 16, with a clinical history of joint instability, recurrent shoulder dislocations, and skin translucency.

Her medical history included postural orthostatic tachycardia syndrome (POTS), bronchial asthma, congenital sensorineural deafness, congenital adrenal hypoplasia, and chronic venous thrombosis. She had a Hickman catheter in place for intravenous hydration and medications due to severe hyperemesis gravidarum. Her medications included prophylactic enoxaparin, prednisolone, bisoprolol, salbutamol, and ondansetron.

Surgical history included two previous cesarean deliveries under neuraxial anesthesia (complicated by postpartum hemorrhage after the first), bilateral XEN® Gel stent insertions for glaucoma, and cochlear implantations. The first CD was upon her request; to prepare for the CD, a multidisciplinary team was formed and developed a tailored perioperative plan (detailed in Discussion).

On the day of surgery, the patient was assessed in the operating theater. She appeared comfortable, alert, and oriented. Baseline vital signs were within normal limits: blood pressure 115/69 mmHg, heart rate 72 beats per minute (regular), and oxygen saturation 97% on room air. Laboratory findings included a hemoglobin level of 11.8 g/dL and a platelet count of 206,000/µL. Coagulation, renal, and hepatic function tests were within normal ranges, and a preoperative echocardiogram showed no abnormalities. Chest auscultation was clear bilaterally, and cardiac examination revealed normal heart sounds. 

Following the initial assessment, intravenous Hartmann’s solution was initiated for co-loading. Due to a documented penicillin allergy, the patient received clindamycin 900 mg as prophylactic antibiotic therapy. Antiemetic and aspiration prophylaxis included ondansetron 6 mg and metoclopramide 10 mg administered intravenously.

**Figure 1:**
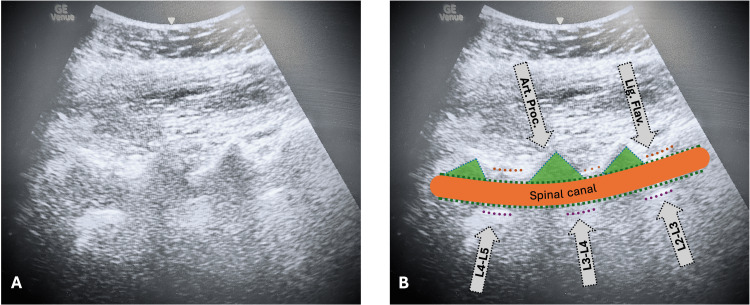
Pre-procedural ultrasound of the lumbar spine to determine the appropriate level for spinal anesthesia. *Panel A *shows the raw ultrasound image obtained in the parasagittal oblique view without annotation. *Panel B* presents the same image with anatomical structures labeled for orientation and procedural planning. The articular processes (Art. Proc.) and ligamentum flavum (Lig. Flav.) are indicated, alongside the estimated location of the spinal canal (orange ellipse). Interspaces between lumbar vertebrae (L2–L3, L3–L4, and L4–L5) are identified to aid in choosing the optimal puncture site. Green shaded areas represent vertebral laminae, while the spinal canal is outlined in orange.

The patient was gently seated on the operating table, and a pre-procedural ultrasound scan of the lumbar spine was performed to identify and mark the L3-L4 interspace (Figure 1). After standard antiseptic preparation and draping, the marked site was anesthetized with 2 mL of 2% lidocaine, and then a 27 G pencil-point spinal needle was introduced using an introducer. Upon confirming free flow of cerebrospinal fluid, a single-shot neuraxial anesthesia was performed with a combination of intrathecal hyperbaric bupivacaine 0.5% 2.2 mL, intrathecal fentanyl 15 mcg, and intrathecal preservative-free morphine 150 mcg (Table 1).

**Table 1:** Intraoperative drug administration timeline. This table summarizes the intraoperative medications administered during cesarean delivery under spinal anesthesia. The timing, dosage, and routes of administration are detailed. Phenylephrine was given as a continuous infusion for blood pressure support. All medications were administered according to standard obstetric anesthesia protocols. This timeline was derived from the original anesthesia record.

Time (HH:MM)	Drug name	Dose	Route	Notes	
08:05	Start anesthesia				
08:06	Clindamycin	900 mg	IV	Antibiotic prophylaxis	
08:07	Ondansetron	6 mg	IV	Antiemetic	
08:07	Metchlopramide	10 mg	IV	Prophylaxis prevention	
08:24	Lidocaine 2%	40 mg	Skin infiltration	Local anesthesia	
	Phenylephrine	100 mcg/ml, 30 ml/hr.	IV	Continous infusion, gradual tappering.	
08:25	Heavy bupivacaine 0.5%	10.5 mg	Spinal	Spinal anesthesia	
	Fentanyl	15 mcg	Spinal	Spinal adjunct	
	Morphine – preservative free	150 mcg	Spinal	Spinal adjunct	
08:39	Started surgery				
08:52				Fetal extraction.	
	Carbetocin	100 mcg	IV	Slow infusion	
	Paracetamol	1 gm	IV	Analgesia	
	Ketorolac	30 mg	IV	Analgesia	
	Dexamethasone	6 mg	IV	Antiemetic, anti-inflammatory, prolongation of spinal analgesia.	
09:17	Traneximic acid	1000 mg	IV	Anti-fibrinolytic	
09:35	End surgery. Blood loss: 650 ml, Hartman’s solution: 750 ml, urine output: 120 ml.				

The patient was carefully repositioned to the supine position, and a phenylephrine infusion was initiated at a concentration of 100 mcg/mL, delivered at a rate of 30 mL/hour to maintain hemodynamic stability. Non-invasive blood pressure (NIBP) was monitored every 2.5 minutes and remained stable throughout the procedure. The phenylephrine infusion was gradually tapered during surgery and discontinued by the end of the procedure, with the patient maintaining stable hemodynamic parameters throughout.

After anesthesia, the sensory block level was assessed every minute using an ice-touch test. A T4 sensory level was confirmed at three minutes, at which point the surgical team was cleared to proceed. The surgery lasted 55 minutes and was uneventful. Then, 750 mL of Hartmann’s solution was administered intravenously, the estimated blood loss was 650 mL, and the urine output was 120 mL; all the position adjustments and transport were smoothly and carefully done due to the patient’s tissue fragility.

Intraoperative analgesia included the intrathecal medications listed above, as well as intravenous ketorolac 30 mg, paracetamol 1 g, and dexamethasone 6 mg, to prolong analgesic duration. Postoperative pain was managed according to the institutional protocol and guided by a visual analog scale (VAS). 

The patient remained in the post-anesthesia care unit (PACU) for three hours under close monitoring, including assessment of hemodynamics, bleeding, urine output, and motor function. She was then transferred to the obstetric ward in stable condition and discharged home on postoperative day 3 with a follow-up plan and emergency contact instructions.

**Figure 2:**
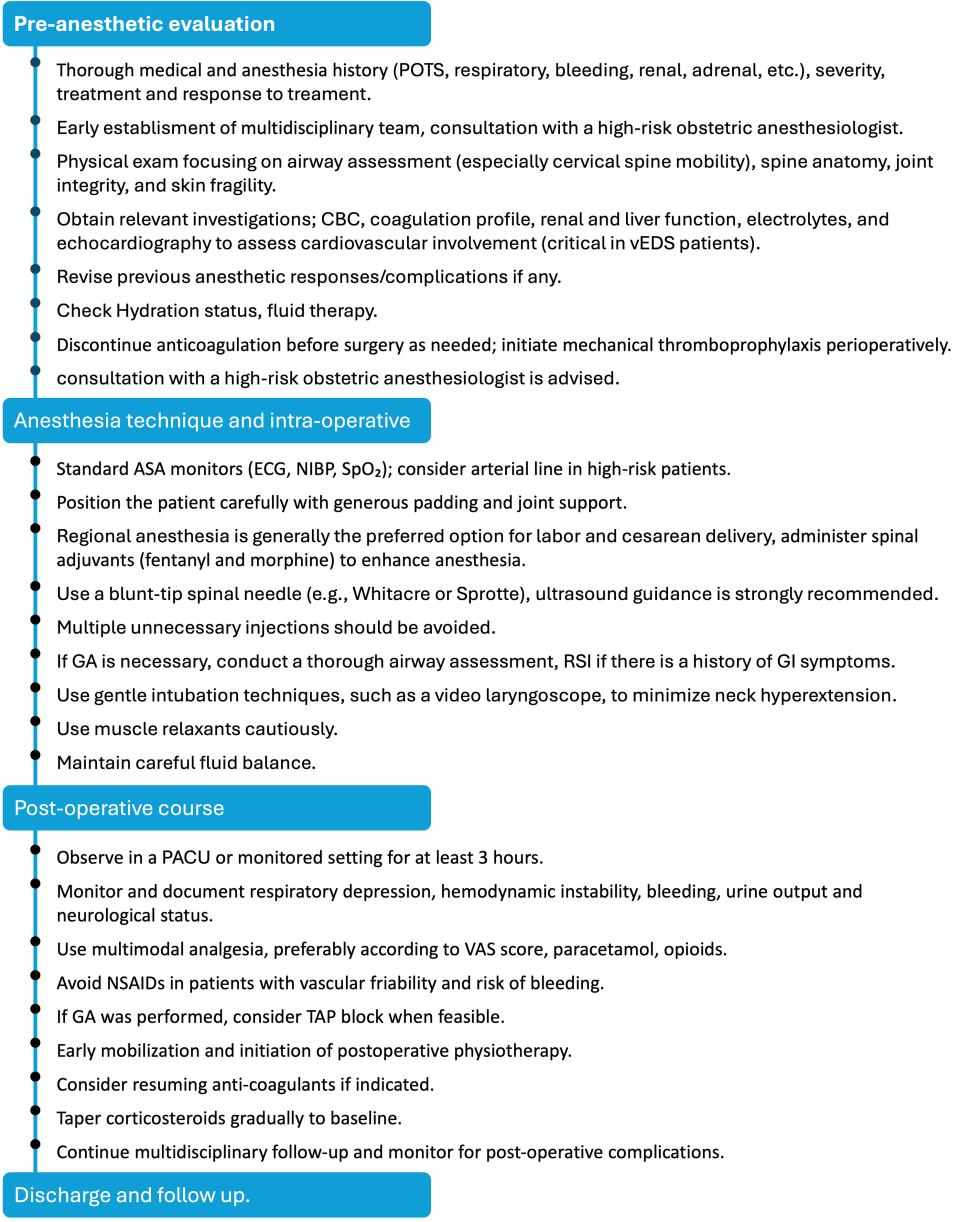
Timeline summarizing anesthetic management in cEDS for caesarean delivery. This figure provides a chronological overview of the patient's clinical course, including pre-anesthetic evaluation, intraoperative anesthetic technique and events, postoperative recovery, and follow-up. Key interventions, monitoring steps, and clinical milestones are summarized to illustrate the perioperative decision-making process. ASA: American Society of Anesthesiologists, CBC: complete blood count, GA: general anesthesia, GI: gastrointestinal, IV: intravenous, NIBP: non-invasive blood pressure, NSAIDs: nonsteroidal anti-inflammatory drugs, PACU: post-anesthesia care unit, POTS: postural orthostatic tachycardia syndrome, TAP: transversus abdominis plane, vEDS: vascular Ehlers-Danlos syndrome

The summarized timeline of the patient's anesthetic and perioperative course is shown in Figure 2.

## Discussion

EDS are a group of inherited connective tissue disorders comprising 13 subtypes, each defined by distinct genetic mutations and clinical features. The most common subtype, hypermobile EDS (hEDS), is characterized by joint hypermobility and chronic pain but lacks a known genetic cause. cEDS presents with skin hyperextensibility and atrophic scarring, while vascular EDS (vEDS) is the most severe, involving fragile blood vessels and a high risk of arterial rupture [6].

The choice of delivery method in EDS patients should be individualized according to the subtype and clinical scenario. Vaginal delivery is often possible in hEDS, whereas cesarean section is generally preferred in vEDS due to the risk of vascular complications [7].

Anesthetic management also requires careful planning. Both general and regional anesthesia have been reported in EDS, but each carries challenges such as tissue fragility, joint instability, and vascular risks. General anesthesia may be complicated by difficult airway, cervical spine instability, temporomandibular joint dislocation, and mucosal fragility [8,9]. In our case, the patient had two prior cesarean deliveries, and we opted for an atraumatic spinal anesthesia technique to minimize tissue damage and avoid airway manipulation.

Although neuraxial anesthesia is generally safe in EDS, resistance to local anesthetics and unpredictable spread of the block have been described [3,4,8]. Given the fragile connective tissue, intraoperative positioning demands careful attention, including generous padding, neutral joint alignment, and avoidance of shear forces. Continuous monitoring and detailed documentation are crucial to detect and manage potential complications promptly [9].

The role of a multidisciplinary team

In this case, coordination among multiple specialties ensured a safe perioperative course:

Anesthesiology opted for spinal anesthesia using ultrasound to reduce the risk of traumatic puncture and spinal hematoma. Given EDS-related resistance to local anesthetics, the team administered a carefully titrated dose of hyperbaric bupivacaine with adjunct opioids. The decision to avoid epidural catheter placement was informed by the potential for catheter misplacement or hematoma in fragile tissues.

Cardiology monitored for POTS-related hemodynamic instability intra- and postoperatively. Although POTS may be exacerbated during pregnancy and anesthesia, this patient remained stable, likely due to optimal fluid management and vasopressor support.

Hematology recommended pausing enoxaparin 24 hours before surgery and resuming thromboprophylaxis postoperatively for six weeks. Given the history of chronic thrombosis and a central line, this plan followed guidelines for high-risk thrombosis patients.

Otorhinolaryngology (ENT) advised using bipolar cautery to prevent electrical interference with cochlear implants, a detail often overlooked but critical in such patients.

Obstetrics modified the surgical technique by using interrupted sutures to reduce tension on fragile connective tissue and applied pressure gauze to minimize postoperative bleeding, aligning with approaches described in previous case reports, and to check for proper wound healing before removing stitches.

Endocrinology managed congenital adrenal insufficiency with stress-dose steroids, an essential intervention during major surgery to prevent adrenal crisis.

Pulmonology ensured asthma control with bronchodilators to avoid perioperative bronchospasm.

Neurology contributed to dysautonomia management by recommending IV thiamine to support autonomic regulation during metabolic stress.

Physiotherapy was initiated early to prevent deconditioning and support safe ambulation.

Vascular surgery was alerted preoperatively and remained on standby, given the patient’s history of venous thrombosis and the theoretical risk of vascular rupture in EDS, especially if there were complications.

This interdisciplinary model aligns with best practice guidelines, which advocate for individualized perioperative planning for EDS patients. Previous reports have shown that a lack of coordination contributes to poor outcomes in such complex cases.

Comparative insights from the literature

While much of the literature on EDS in pregnancy centers on hypermobile or vascular subtypes, this case highlights the unique anesthetic considerations in cEDS. For instance, Jones et al. described a case of cesarean section in EDS with POTS under general anesthesia and emphasized the risk of hemodynamic lability. By contrast, our use of neuraxial anesthesia with active monitoring and phenylephrine infusion contributed to stable intraoperative vitals [5].

Moreover, reports by Wiesmann et al. and Laserna et al. highlighted unpredictable block levels and anesthetic resistance in EDS [3,4,10,11]. Our case corroborates this with careful titration and confirmation of block height via ice testing. No resistance was encountered, but awareness of this possibility shaped drug selection and monitoring strategy.

Lastly, delayed wound healing and postoperative dehiscence have been observed in EDS due to collagen abnormalities [7]. The surgical team's adjustment of suture technique and wound pressure dressing likely contributed to the uneventful postoperative recovery in our patient.

## Conclusions

The anesthetic management of pregnant patients with EDS requires a highly individualized, multidisciplinary approach due to the complex interplay of tissue fragility, cardiovascular instability, and comorbid conditions. This case illustrates the successful use of ultrasound-guided spinal anesthesia with extended post-anesthesia monitoring in a patient with cEDS and multiple systemic comorbidities. Tailored perioperative planning, meticulous technique, and collaboration across specialties are essential to optimize maternal and fetal outcomes in this high-risk population.
